# New phenotype of DCTN1‐related spectrum: early‐onset dHMN plus congenital foot deformity

**DOI:** 10.1002/acn3.50985

**Published:** 2020-02-05

**Authors:** Wo‐Tu Tian, Li‐Hua Liu, Hai‐Yan Zhou, Chao Zhang, Fei‐Xia Zhan, Ze‐Yu Zhu, Sheng‐Di Chen, Xing‐Hua Luan, Li Cao

**Affiliations:** ^1^ Department of Neurology Rui Jin Hospital Shanghai Jiao Tong University School of Medicine Shanghai China; ^2^ Department of Neurology Jurong Hospital Affiliated to Jiangsu University Jurong People’s Hospital Jurong Jiangsu Province China

## Abstract

**Objective:**

To describe the clinical and genetic features of two patients with different phenotypes due to various Dynactin 1 (DCTN1) gene mutations and further explore the phenotype–genotype relationship.

**Methods:**

Patient 1 is a 23‐year‐old man with congenital foot deformity and life‐long distal muscle weakness and atrophy. Patient 2 is a 48‐year‐old woman with adult‐onset progressive weakness, lower limbs atrophy, and pyramid bundle signs. Electrophysiology test showed normal nerve conduction velocity of both patients and neurogenic changes in needle electromyography. Open sural nerve biopsy for Patient 1 showed slight loss of myelinated nerve fibers. Both patients were performed with whole‐exome sequencing followed by functional study of identified variants.

**Results:**

Two mutations in *DCTN1* gene were identified in Patient 1 (c.626dupC) and Patient 2 (c.3823C>T), respectively. In vitro, the wild type mostly located in cytoplasm and colocalized with α‐tubulin. However, c.626dupC tended to be trapped into nuclear and the c.3823C>T formed cytoplasmic aggregates, both losing colocalization with α‐tubulin. Western blotting showed a truncated mutant with less molecular weight of c.626dupC was expressed.

**Interpretation:**

We identify two novel *DCTN1* mutations causing different phenotypes: (1) early‐onset distal hereditary motor neuropathy plus congenital foot malformation and (2) amyotrophic lateral sclerosis, respectively. We provide the initial evidence that foot developmental deficiency probably arises from subcellular localizing abnormality of Dynactin 1, revealing *DCTN1*‐related spectrum is still expanding.

## Introduction

Dynactin 1 (DCTN1), the largest subunit of the dynactin complex, interacts with microtubule and tubulin dimer, plays a protective role in stabilizing neuron cytoskeleton, and initiates dynein‐mediated axonal retrograde transport.^1^, ^2^ *DCTN1*‐related axonal deficiency leading to inherited motor neuron degeneration, ranges widely from distal hereditary motor neuronopathy (dHMN), Perry syndrome to amyotrophic lateral sclerosis (ALS).^3^, ^4^, ^5^ Different phenotypes are mainly characterized as involvement of inferior motor neurons or superior motor neurons or both, with different ages of onset and various progress speed. *DCTN1*‐related dHMN, also known as dHNM type 7B, presents with distal limb dystrophy and weakness appearing at early adulthood,^6^ whereas Perry syndrome is characterized by middle‐age‐onset parkinsonism, depression, weight loss, and central hypoventilation.^4^, ^7^, ^8^ Recently, frontotemporal dementia (FTD) has also been considered as one of the phenotypes,^9^ indicating the *DCTN1*‐related spectrum is still expanding. Since the year of 2003, *DCTN1* mutation was first associated with human disease,^6^ more than 20 variations have been reported in sporadic or familial cases.^4^, ^5^ However, rare of the *DCTN1* mutations have been functionally examined and the pathogenesis remains elusive.

Here we identified two patients due to different novel *DCTN1* mutations, manifesting as very early‐onset dHMN plus congenital foot deformity and ALS, respectively. On the basis of thorough clinical, pathological, and genetic analysis, we aimed to functionally investigate the pathogenesis of *DCNT1*‐related spectrum.

## Materials and Methods

### Participants

We identified two patients fulfilling the diagnosis of motor neuron disease according to distal muscle atrophy and weakness as well as electrophysiological evidence.^10^, ^11^ Two patients and their parents were clinically examined.

### Standard protocol approvals, registrations, and patient consents

The ethics committee of Rui Jin Hospital affiliated to Shanghai Jiao Tong University School of Medicine approved the study. All participants provided written informed consent.

### Mutation analysis

Genomic DNA was extracted using a standard phenol/chloroform extraction protocol. Healthy individuals (*n* = 300) of matched geographic ancestry were included as normal controls. Exome sequencing was performed for the patients, using Agilent SureSelect v6 reagents for capturing exons and Illumina HiSeq X Ten platform. Alignment to human genome assembly hg19 (GRCh37) was carried out followed by recalibration and variant calling. Population allele frequencies compiled from public databases of normal human variation (dbSNP, ESP6500, and 1000 g) were used to initially filter the data to exclude variants at greater than 1‰ frequency in the population. The variants were further interpreted and classified according to the American College of Medical Genetics and Genomics (ACMG) Standards and Guidelines.^12^ In this segment, two neurogeneticists analyzed the inheritance pattern, allele frequency (from: 1000 g, ESP6500, dbSNP, ExAC, and 300 in‐house ethnically matched healthy controls), amino acid conservation, pathogenicity prediction [Mutationtaster (http://www.mutationtaster.org)]. Putative pathogenic variants were further confirmed by Sanger sequencing both forward and reverse strands.

### Neuropathology

We performed open biopsy on the left sural nerve in Patient 1. The peripheral nerve tissue was frozen and then cut at 7 μm sections. These sections were stained according to standard histological and histochemical procedures with hematoxylin and eosin (HE), modified Gomori Trichrome (MGT), and Congo Red.

### Electron micrograph

Fresh nerve tissue was postfixed in 2% PFA/2.5% glutaraldehyde in phosphate buffer, pH 7.2 overnight at 4°C. After PBS buffer rinse, samples were postfixed in 1% osmium tetroxide buffer (2 h) on ice in the dark. After a double‐distilled water rinse, tissue was stained with 3% aqueous uranyl acetate (0.22‐μm filtered; 1 h in the dark), dehydrated in a graded series of ethanol and propylene oxide, and embedded in Epoxy 618 resin. Samples were polymerized at 60°C for 48 h. Toluidine blue staining was performed on semithin sections (0.7–1.0 μm). Thin sections (60–90 nm) were cut with a diamond knife on the LKB V ultramicrotome and picked up with formvar‐coated copper slot grids. Grids were stained with lead citrate and observed with transmission microscopy (PHI LIP CM‐120).

### Cell culture, transfection, and western blotting

HEK 293T cells were obtained from the Cell Bank of Chinese Academy of Sciences (http://www.cellbank.org.cn) and maintained in Dulbecco's Modified Eagle Medium (DMEM) with 10% fetal bovine serum (FBS) and 1% penicillin/streptomycin (PS) at 37°C in a humidified incubator with 5% CO_2_. One day before transfection, cells were plated at 150,000 cells per well in 6‐well culture dish. The next day, cells were transfected with 2.5 μg of EGFP control plasmid DNA or DCTN1‐EGFP wild‐type (DCTN1‐WT) or mutant (c.626dupC and c.C3823T) plasmid DNA using Lipofectamine 3000 transfection reagent (Invitrogen). Forty‐eight hours later, the cells were split in radio immunoprecipitation assay (RIPA) buffer (Beyotime) to extract protein for western blot analysis. Cell lysates were diluted in 6X SDS‐PAGE Sample Loading Buffer (Beyotime) for protein denaturation. For cell lysates, equal volumes were run on 8% SDS polyacrylamide gels. Total DCNT1 levels were detected using the anti‐GFP antibody (1:2500, GFP‐1010, AVES). GAPDH primary antibody (1:1000, 2118‐14C10, Cell Signaling Technology) or Histone H3 primary antibody (1:2000, D1H2, Cell Signaling Technology) were used to ensure equal protein loading. Blots were then incubated with anti‐chicken and anti‐rabbit secondary HRP‐conjugated antibodies (1:5000, Beyotime) and bands were detected by enhanced chemiluminescence using Western Blot Enhancer reagents (Thermo Scientific).

Fibroblasts from Patient 1 and control individual were obtained from skin biopsies after informed consent. Fibroblasts were maintained at 37°C in a humidified incubator containing 5% CO2 in Dulbecco's modified Eagle's medium supplemented with 10% calf bovine serum and 0.1% gentamycin. Protein of fibroblast cell lysate was extracted and the expression level of DCTN1 was detected by western blotting with mouse anti‐p150 Glued (1:1000, 610473, BD Transduction Laboratories) or goat anti‐DCTN1 (1:1500, ab11806, Abcam).

### Primary neuron culture

Primary cell culture of cortex neurons was performed as described.^13^ Briefly, cortex neurons were dissociated from P0 pups. The triturated cells (1 × 10^5^ cells per well) were grown on glass coverslips coated with 10 μmol/L poly‐lysine overnight in 24‐well dishes. Then the culture was grown in a medium of Neurobasal A media (Gibco) supplemented with B27 and 2 mmol/L glutamine for the indicated number of days. The cortex neurons were transfected with DCTN1‐WT/Mut plasmid at DIV7 using the calcium phosphate method.

### Immunofluorescence

HEK 293T cells and primary neuron transfected with the respective expression constructs were washed in PBS and fixed using 4% paraformaldehyde for immunofluorescence test. Cells were blocked with 10% normal donkey serum and 0.3% Triton X‐100 in PBS for 60 min, incubated with primary antibody (GFP‐1010, AVES; α‐Tubulin‐11H10, CST) in blocking solution at 4°C overnight and incubated with Alexa Fluor 488 or 594 secondary antibodies (1:1000, Life). DAPI (4′,6‐diami‐dino‐2‐phenylindole) (1:10,000, Life) was used for nucleic acid staining. Images were taken with a Zeiss 710 confocal microscope.

### Data availability statement

The original dataset used and analyzed for this study is available from the corresponding author on reasonable request.

## Results

### Clinical findings

**Figure 1:**
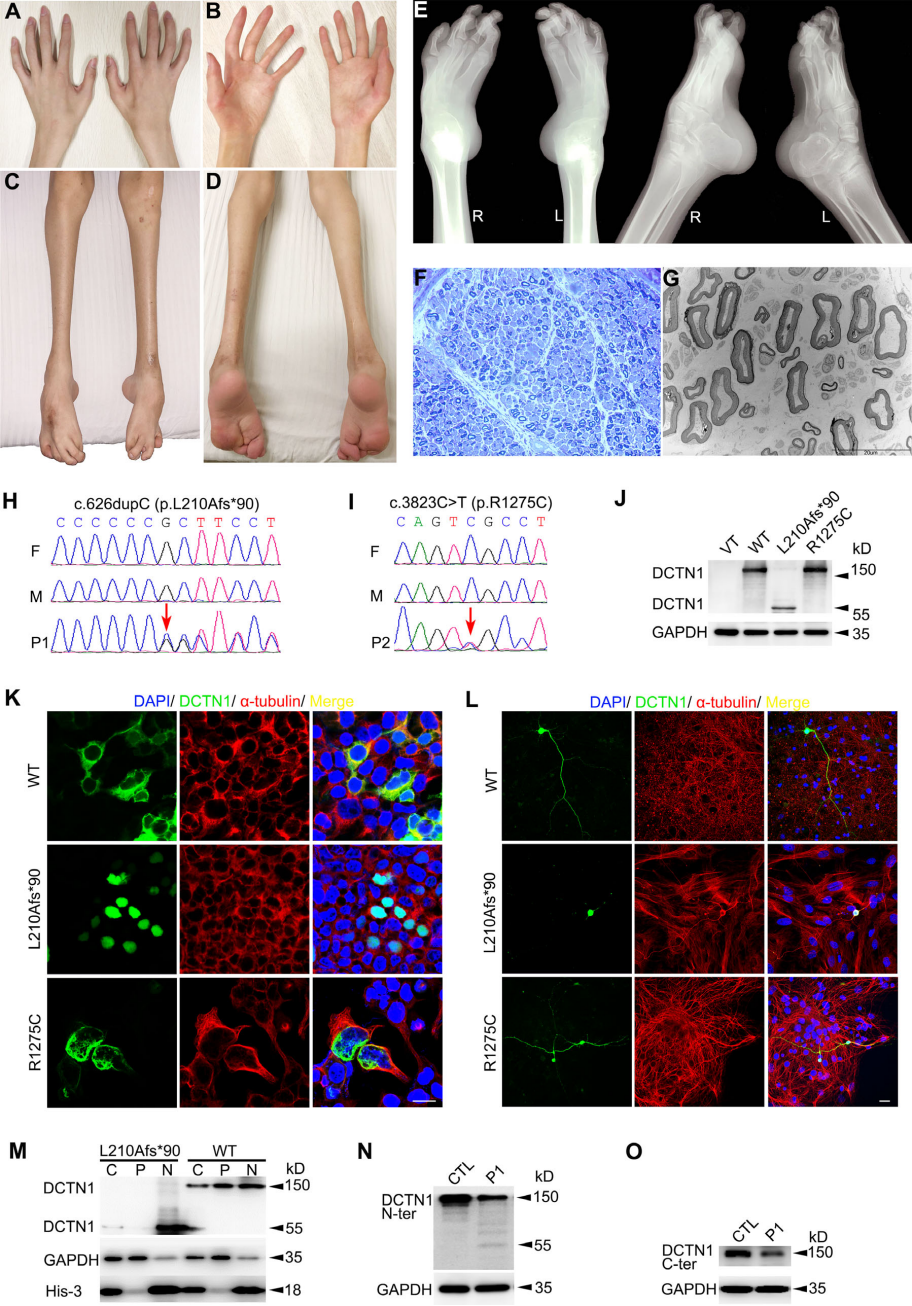
Clinical, pathological, genetic, and functional characterizations. (A–D) Hands (A and B) and lower limbs (C and D) of Patient 1. (E) X‐ray photograph of lower limbs of Patient 1 showing bilateral talipes equinovarus. (F and G) Peripheral nerve pathology of the Patient 1 showed mild density decrease of large myelinated fibers by toluidine blue staining (F) and under electronic microscopy (G). (H) Sequence chromatograms of *DCTN1* gene of Patient 1’s family. It displays one de novo insertion mutation of c.626dupC (arrow) in the proband. F = father, M = mother, P1 = patient 1. (I) Sequence chromatograms of *DCTN1* gene of Patient 2’s family. It displays one de novo missense mutation of c.3823C>T (arrow) in the proband. (J) Western blotting showed the expressions of DCTN1‐Mut (L210Afs*90) were significantly lower than healthy control. Western blotting showed the DCTN1‐WT and R1275C with molecular weight of 150 kDa. DCTN1‐L210Afs*90 with relative smaller molecular weight (∼55 kDa) was detected. (K) DCTN1‐WT/Mut‐EGFP transfected HEK 293T cells showing the presence of WT in cytoplasmic distribution colocalizing with α‐tubulin but L210Afs*90 is expressed in nuclear and R1275C forms punctate aggregates. The scale bar represents 20 μm. (L) Immunofluorescence of DCTN1‐WT/Mut in primary mouse cortex neuron showing WT and R1275C is expressed in both body and axon, but L210Afs*90 is only expressed in the body. The scale bar represents 20 μm. (M) Western blotting of separated whole cell/nuclear/cytoplasmic components showed the L210Afs*90 is mainly expressed in nuclear. GAPDH and His‐3 are set as housekeeping proteins. C = cell, N = nuclear, P = cytoplasmic. (N–O) Western blotting showed a strong signal at ~150 kDa of skin fibroblasts from control samples. There was relative weak signal detected in the Patient 1’s protein using either N‐terminus (N) or C‐terminus antibody (O). A signal at relative smaller molecular weight (~55kDa) was detected in the patient but not in control using N‐ terminus antibody (N).

Patient 1 (T3188) is a 23‐year‐old man with lifelong weakness of distal limbs, abnormal gait, and congenital foot malformation. He was born of full‐term spontaneous vaginal delivery with normal birth weight (3.1 kg). Bilateral clubfoot was observed at birth. He achieved sitting and standing at 10 months and 2 years old, respectively. At the age of three years, he learned to walk alone but was unstable, with falling every 5–6 steps. Orthotics surgeries were performed on feet twice at the age of 1 and 6 years, respectively. Hands are clumsy since childhood, especially for delicate manipulating. At the age of 16 years, the muscle twitching on both thighs appeared. Wearing the orthopedic shoes, he may walk slowly and vacillatingly. However, distal dominant limb weakness and muscle atrophy slowly progressed without any sensory disturbances. At the age of 23 years, he was 180 cm in height with body weight 52 kg, clear in speech and alert in recognition. Upon evaluation, tongue fasciculations was continuous when stuck out. He had normal strength in neck flexion and proximal limbs (5/5 rated on a scale of 0/5–5/5), but reduced strength of grip (4/5). Muscle tone in four limbs was normal. Fingers could not be straightened with limited interphalangeal joint extension, obvious interosseous, and thenar muscle atrophy (Fig. 1A and B) as well as postural tremor. Muscle atrophy below knee was eye‐catching. Foot deformity was composed of Achilles tendon contracture as well as toe varus (Fig. 1C and D). Tendon reflexes were brisk in four limbs. Patellar clonus was positive on the right side. The pathological plantar reflex examination was not completed due to unsatisfactory cooperation. Ptosis, nystagmus, dysarthria, dysphagia, ataxia, muscle pain, and dyspnea were not noticed. Mental psychological and cognitive tests were normal. He has been training in backstroke team for six years mainly depending on arm strength. The electrophysiologic showed nerve conduction velocity was normal. Compound muscle action potential (CMAP) was decreased in nervus peroneus communis (0.96 mV), median nerve (1.27 mV), and ulnar nerve (4.4 mV). The duration and amplitude of motor unit potential (MUP) and multiphase potentials were increased, without spontaneous activity, indicating extensive chronic neurogenic lesions. Figure 1E showed the X‐ray photography of distal extremities at the age of 23 years.

Patient 2 (T3658) is a 46‐year‐old woman with progressively worsened weakness and muscle atrophy for 2 years. Initially, weakness affected the right lower limb, leading to occasional falls during walking. Gradually, both lower limbs and hands got involved with interosseous muscle atrophy. Meanwhile, the sensation of muscle twitching developed all over the body. She became incapable of looking after herself and walking alone within only one year, with obvious weight loss, swallowing difficulty, and breath shortness. The parameters of postnatal and adolescent development did not provide any clue of growth abnormality. However, she had constipation for more than 30 years. Upon physical examination, she presented reduced fluency of speech and tongue fasciculations. She had reduced strength in neck flexion (1/5), distal upper limbs (3/5), and lower limbs (4/5), but normal strength in proximal upper limbs (5/5). Atrophy of interosseous and thenar muscles. Muscle tone in four limbs was normal. Tendon reflexes were brisk in four limbs with persistent patellar clonus and ankle clonus. The pathological plantar reflex was negative. Mental psychological and cognitive tests were in normal range. The electrophysiologic examination showed decreased motor nerve conduction velocity and CMAP amplitude, with fibrillation and positive sharp waves, widened MUP upon light contraction, with or without multiphase potential.

### Neuropathological findings

Sural nerve biopsy was performed on Patient 1, without significantly morphological changes by HE, MGT, Congo Red staining. Myelinated fibers of large diameter are slightly decreased in density by toluidine blue staining (Fig. 1F) or under electronic microscopy (Fig. 1G).

### Genetic findings

Patient 1 was identified with a de novo frameshift heterozygous variant c.626dupC (p.L210Afs*90) in *DCTN1* gene (Fig. 1H). Patient 2 was identified with a de novo missense heterozygous variant c.C3823T (p.R1275C) in the same gene (Fig. 1I). Both variants were not identified in the patients’ parents, 300 healthy controls, dbSNP (http://www.ncbi.nlm.nih.gov/snp), 1000 Genome Project (http://browser.1000genomes.org), NHLBI Exome Sequencing Project (ESP) Exome Variant Server (http://evs.gs.washington.edu/EVS) or Exome Aggregation Consortium (ExAC). p.L210Afs*90 was predicted to be disease causing by Mutationtaster (probability score 1.000). p.R1275C was predicted to be probably damaging by PolyPhen2 (probability score 0.997, sensitivity: 0.41, specificity: 0.98), damaging by SIFT (SIFT score: 0.000), and disease causing by Mutationtaster (probability score 0.999).

### Mutant protein detection

The expression of DCTN1‐WT/Mut was examined by western blotting after transfecting HEK 293T cells with DCTN1 constructs, showing the p.L210Afs*90 with smaller molecular weight than WT (Fig. 1J). In immunofluorescence staining (Fig. 1K), the p.L210Afs*90 completely overlapped with DAPI‐stained nucleus in comparison with the cytoplasmic distribution of WT. The cytoplasmic colocalization of DCTN1‐WT and α‐tubulin was confirmed. However, R1275C tended to form punctate aggregates, partially losing colocalization with α‐tubulin. Furthermore, to figure out how the mutant protein acted in primary neuron, we transfected DCTN1‐WT/Mut into mouse cortex neuron. Both WT and R1275C distributed throughout the neuron both body and axon (Fig. 1L). p.L210Afs*90 could not reach out to the axon and mainly expressed in the body (Fig. 1L). In western blotting, p.L210Afs*90 was almost exclusively expressed in the separated nucleus lysate (Fig. 1M). We further tested the expression of DCTN1 in Patient 1’s skin fibroblasts. A truncated smaller DCTN1 was tested at around 55kD using anti‐DCTN1 N‐ter antibody (Fig. 1N). Using anti‐DCTN1 C‐ter antibody, we can see the expression of DCTN1 of whole length in Patient 1 was half of healthy control via three repeated tests (Fig. 1O).

## Discussion

We described two sporadic patients with motor neuron disease due to de novo mutations in *DCTN1*, c.626dupC, and c.C3823T, respectively. Patient 2 manifested with typical features of ALS. However, Patient 1 with distal motor neuropathy had an extremely early age of onset as well as congenital foot deformity, in comparison with all other DCTN1‐related cases reported before. Interestingly, *DCNT1* mutation can also lead to Perry syndrome, which is characterized by rapidly progressive parkinsonism accompanied by depression/apathy, unintentional weight loss and respiratory failure.^4^, ^8^, ^14^ Patients usually show levodopa‐resistant/responsive resting tremor, rigidity, bradykinesia, postural instability and autonomic dysfunction, and reminiscent of Parkinson’s disease. Therefore, Perry syndrome can be easily misdiagnosed as Parkinson’s disease, especially during early stage. The major death cause is central hypoventilation or pneumonia.^8^, ^15^, ^16^, ^17^, ^18^ The clinical characterizations of DCNT1‐related spectrum are summarized in Table 1.

**Table 1:** Clinical characterizations of *DCTN1*‐related spectrum.

Phenotype	dHMN	ALS	Perry syndrome
Onset	Early adulthood or earlier	48–64 yrs	35–70 yrs
Disease duration	Slow progression	4–9 yrs	≤5 yrs
Initial symptoms	Hand weakness	Hand and lower limb weakness	Depression/apathy, parkinsonism, weight loss, central hypoventilation
Cardinal symptoms	Face, distal limb weakness and muscle atrophy	Progressive limb weakness and muscle atrophy, increased muscle tone, tendon hyperreflexia, positive pathological sign	Parkinsonism, depression/apathy, respiratory symptoms, weight loss
Bulbar symptoms	Vocal cord paralysis, shortness of breath	Vocal cord spasm, difficulty speaking, difficulty swallowing	Dysphasia, difficulty swallowing
Other clinical features	Foot deformity	Constipation, severe unintentional weight loss, frontotemporal dementia	Cognitive impairment, sleep disturbances
Cause of death	NA	Respiratory failure	Respiratory failure/pneumonia
Motor neuron involved	Distal motor neuropathy	Both upper and lower motor neuron	Upper motor neuron

dHMN = distal hereditary motor neuropathy, ALS = amyotrophic lateral sclerosis, yrs = years, NA = not available.

The *DCTN1* gene encodes Dynactin subunit 1, the biggest subunit among all 10 subunits of Dynactin complex. P150^Glued^ is encoded by full‐length *DCTN1* gene, weights around 150 kD and contains the N‐terminal cytoskeleton‐associated protein glycine‐rich (CAP‐Gly) domain, followed by two coiled‐coil domains (CC1 and CC2).^1^ The CAP‐Gly domain (aa 48–90) has a ^(67)^GKNDG^(71)^ motif, which is evolutionarily conserved and plays a critical role in microtubule binding.^1^, ^19^, ^20^ Dynactin 1 is essential in enriching dynactin at neurite tips through the end‐binding proteins (EB1 and EB3), which are both microtubule‐binding molecular.^21^ Dynactin accumulation enhances recruitment and sustained engagement of dynein.^22^, ^23^ It is also required for the dynein‐driven retrograde flux of organelles and vesicles along microtubules from distal axon.^2^, ^24^, ^25^

**Figure 2:**
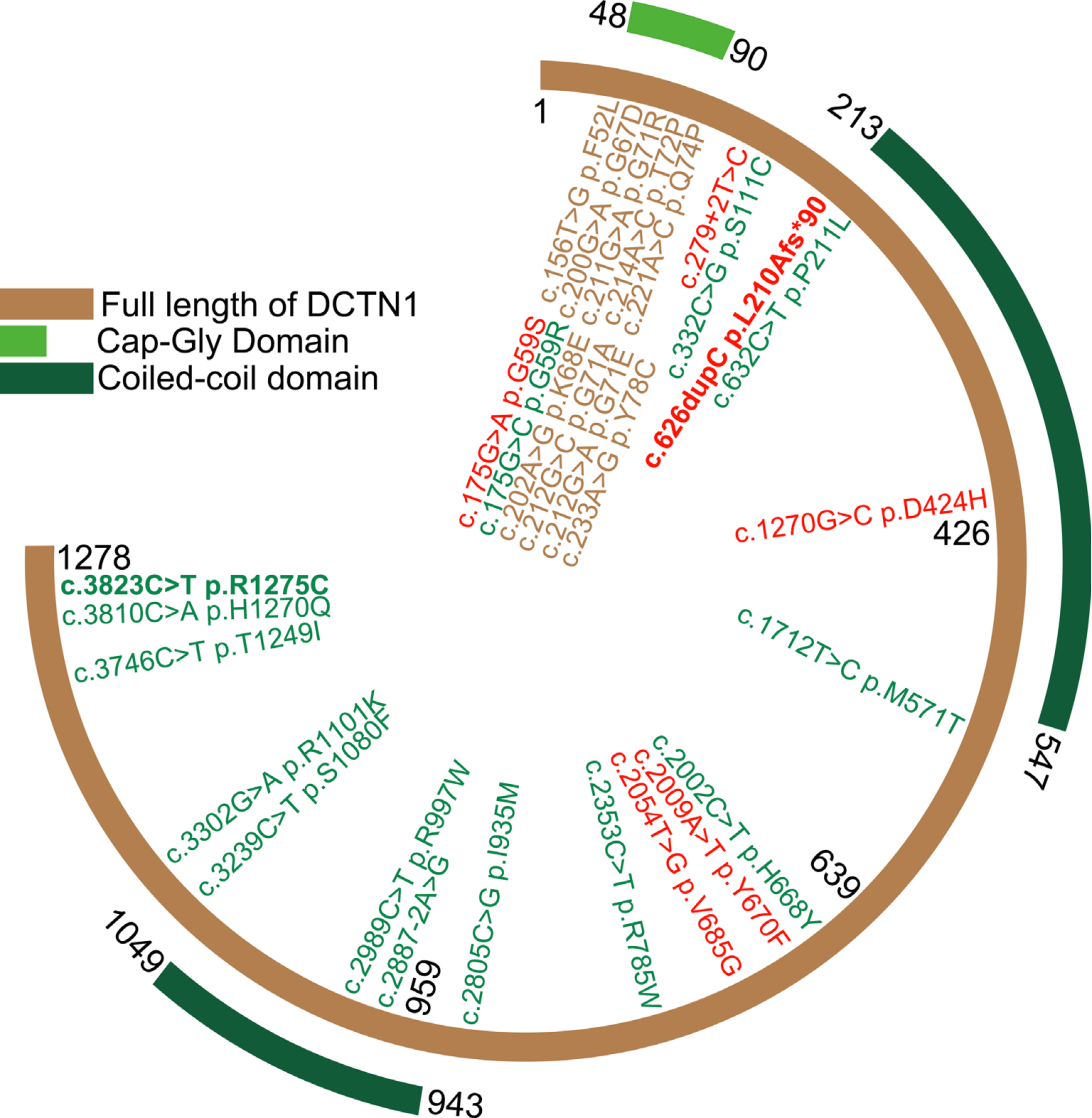
The schematic diagram of DCTN1 structure with mutations. Full length of DCNT1 (NM_004082) in brown consists of 1278 amino acids. Mutations identified with dHMN, ALS, and Perry syndrome are in red, green, and brown, respectively. Mutations firstly identified in this paper are in bold font, c.626dupC (p.L210Afs*90) and c.C3823T (p.R1275C).

So far, a total of 29 mutations in *DCTN1* have been associated with diseases, including 26 missense, 1 frameshift, and 2 splicing variants (Fig. 2). Interestingly, all the Perry syndrome‐related mutations map to CAP‐Gly domain (9/9, Fig. 2, brown), among which G71A, G71R, G71E, T72P, and Q74P affect the EB1 interaction and the stability of CAP‐Gly domain.^26^ Moreover, most of the ALS‐related mutations are more likely to locate on C‐ter half (10/14, Fig. 2, green). Among these, several have been confirmed to result in lower DCTN1 protein expression.^27^, ^28^ c.C3823T (p.R1275C) found in Patient 2 with ALS, lies within the domain (aa 911–1278) interacting with Hermansky‐Pudlak syndrome 6 protein (HPS6).^29^ The R1275C appearing as cytoplasmic aggregates, completely lost colocalization with microtubules. In the cytoplasm, organelle transport from cell periphery to perinuclear site is dependent on the interaction between microtubule networks and dynein/dynactin complex, such as the centripetal movement of endosomes and autophagosomes.^1^

There are another six mutations identified in patients with lower motor neuron disease (Fig. 2, red), among which, DCTN1‐G59S has been confirmed with weakened affinity with microtube.^6^ To be noted, DCTN1‐related HMN reported previously are all adult‐onset. Nevertheless, c.626dupC (p.L210Afs*90), the only frameshift mutation, is identified in our patient with congenital bilateral clubfoot, delay in motor milestones, and neonatal‐onset lower motor neuron disease. p.L210Afs*90 is located in front of the first coiled‐coil domain (CC1), responsible for binding the dynein intermediate chain.^30^, ^31^ The frameshift results in changes of a series of amino acids downstream and introduces a premature stop codon, leading to 979 missing amino acids (76.6% of the whole length). At the N‐ter of DCTN1, aa ^(148)^RRPKP^(152)^ is a nuclear localization signal (NLS) tagging the protein to be imported into cell nucleus. Besides, aa ^(227)^LEEKLETLRL^(236)^ is a nuclear export signal (NES) targeting the protein to be exported from cell nucleus to the cytoplasm. The frameshift occurred between NLS and NES, leading to NLS remained but NES missed. Consistently, we find the truncated Dynactin 1 protein trapped into the nuclear thus losing right cytoplasmic localization and interaction with microtubule. In Western blotting, the whole‐length band from Patient 1 is weaker than the control’s, and the band of truncated mutant (about 55kDa) is even weaker, indicating the mutant protein might be recognized by protein degradation pathway. Consequently, c.626dupC (p.L210Afs*90) is mostly likely to cause haploinsufficiency due to frame‐shift caused loss‐of‐function.^32^ During mitosis, microtubules are required to control the rearrangements of the nuclear membranes.^33^ Especially in G2/M transition, nuclear membrane surrounding chromosomes in interphase should be replaced by cytoplasmic spindle microtubules.^34^, ^35^ p150^Glue^, localizing in the nuclear envelope during prophase, plays an essential role in nuclear envelope breakdown (NEBD).^36^ Dynein/dynactin is required for the mitosis as germline deletion of p150^Glued^ causes early embryonic lethality and apoptosis in p150^Glued^ knockout mice.^37^ The depletion of p150^Glued^ induces severe cell‐cycle block before mitosis, due to the lack of NEBD.^36^ Similar phenotypes have also been observed in another cell‐cycle regulation gene, *VRK1* (vaccinia‐related kinase 1), including dHMN, ALS, and spinal muscular atrophy (SMA).^38^, ^39^

P150^Glued^ is essential in maintaining microtubule stability by acting as an anticatastrophe factor in neuronal cultures.^40^ However, we did not observe any substantial loss of axons in peripheral nerve biopsy for Patient 1. Similar findings were convinced in p150^Glued^ cKO mice, where the increased acetylation of α‐tubulin may serve as a compensatory mechanism to maintain the microtubule integrity in the absence of p150Glued.^41^ During fetal development, limbs may fail to grow properly due to congenital or acquired factors.^42^, ^43^ However, Patient 1’s mother was never exposed to poisonous or pernicious environment during pregnancy. In addition, the genetic study does not reveal mutations in known genes associated with congenital club foot.^44^ As the relationship between limb and nervous system development discussed before,^45^, ^46^ patients with hereditary neuropathies may present with early or congenital foot deformities and variable delay in motor milestones due to several genes, such as *FGD4*, *PRX*, *MTMR2*, *SBF2*, *SH3TC2*, *GDAP1* mutations, and Charcot–Marie–Tooth type 1A (CMT1A) duplication.^47^ In this study, the early‐onset dHMN plus congenital foot deformity is probably related with the large fragment deletion of DCTN1 as well as lose of appropriate cytoplasmic localization.

Both ALS and Perry syndrome are rapidly progressing and even leading to death within 4–9 years, whereas the progress of dHMN is relatively slow.^4^ Although the genotype–phenotype correlation can be partially summarized (Fig. 2), the specific mechanisms by which different mutations lead to different phenotypes remain unclear.

## Conclusion

This work reported new phenotype of *DCTN1*‐related spectrum and identified two novel *DCTN1* mutations causing different phenotypes, early‐onset dHMN plus congenital foot malformation and ALS. The mutations lead to abnormal Dynactin 1 distribution, losing the colocalization with microtubule. This study provides the initial evidence that truncated Dynactin 1 trapped within nuclear and can be related with lower limb development abnormality and an extremely early‐onset form of dHMN. Thus, we suggest that *DCTN1*‐related spectrum is still expanding.

## Conflict of Interest

Tian is in charge of Doctoral Innovation Fund of Shanghai Jiao Tong University School of Medicine (No. BXJ201913). Zhou is in charge of Natural Science Foundation of Science and Technology of Shanghai (15ZR1426700). SD Chen is in charge of National Natural Science Foundation of China (81430022). Luan is in charge of National Natural Science Foundation of China (81200965), the Research Fund for the Doctoral Program of Higher Education (20110073120088), and Guang Ci Qing Nian Grant (GCQN‐2017‐A03). L Cao is in charge of National Natural Science Foundation of China (81271262 and 81571086), Shanghai Municipal Education Commission‐Gaofeng Clinical Medicine Grant (20161401), and Interdisciplinary Project of Shanghai Jiao Tong University (YG2016MS64). The other co‐authors report no disclosures relevant to the manuscript.

## Data Analysis

Wo‐Tu Tian, Li Cao, Department of Neurology, Rui Jin Hospital, Shanghai Jiao Tong University School of Medicine, Shanghai, China.

## Author Contributions

Tian: funding, data acquisition, analysis and interpretation of data, study design, statistical analysis, drafting the manuscript. Liu: data acquisition, analysis and interpretation of data, statistical analysis. Zhou, Zhang, Zhan, Zhu: data acquisition. SD Chen, Luan, L Cao: funding, study design and conceptualization, data acquisition, analysis and interpretation of data, manuscript revision.

## References

[1] Schroer TA . Dynactin. Annu Rev Cell Dev Biol 2004;20:759–779. 15473859 10.1146/annurev.cellbio.20.012103.094623

[2] Ayloo S , Lazarus JE , Dodda A , et al. Dynactin functions as both a dynamic tether and brake during dynein‐driven motility. Nat Commun 2014;5:4807. 25185702 10.1038/ncomms5807 PMC4470572

[3] Levy JR , Holzbaur EL . Cytoplasmic dynein/dynactin function and dysfunction in motor neurons. Int J Dev Neurosci 2006;24:103–111. 16406469 10.1016/j.ijdevneu.2005.11.013

[4] Konno T , Ross OA , Teive HAG , et al. DCTN1‐related neurodegeneration: Perry syndrome and beyond. Parkinsonism Relat Disord 2017;41:14–24. 28625595 10.1016/j.parkreldis.2017.06.004 PMC5546300

[5] Münch C , Sedlmeier R , Meyer T , et al. Point mutations of the p150 subunit of dynactin (DCTN1) gene in ALS. Neurology 2004;63:724–726. 15326253 10.1212/01.wnl.0000134608.83927.b1

[6] Puls I , Jonnakuty C , LaMonte BH , et al. Mutant dynactin in motor neuron disease. Nat Genet 2003;33:455–456. 12627231 10.1038/ng1123

[7] Farrer MJ , Hulihan MM , Kachergus JM , et al. DCTN1 mutations in Perry syndrome. Nat Genet 2009;41:163–165. 19136952 10.1038/ng.293 PMC2813485

[8] Mishima T , Fujioka S , Tomiyama H , et al. Establishing diagnostic criteria for Perry syndrome. J Neurol Neurosurg Psychiatry 2018;89:482–487. 29089398 10.1136/jnnp-2017-316864 PMC5909757

[9] Münch C , Rosenbohm A , Sperfeld AD , et al. Heterozygous R1101K mutation of the DCTN1 gene in a family with ALS and FTD. Ann Neurol 2005;58:777–780. 16240349 10.1002/ana.20631

[10] Douglass CP , Kandler RH , Shaw PJ , McDermott CJ . An evaluation of neurophysiological criteria used in the diagnosis of motor neuron disease. J Neurol Neurosurg Psychiatry 2010;81:646–649. 20522872 10.1136/jnnp.2009.197434

[11] Rossor AM , Kalmar B , Greensmith L , Reilly MM . The distal hereditary motor neuropathies. J Neurol Neurosurg Psychiatry 2012;83:6–14. 22028385 10.1136/jnnp-2011-300952

[12] Richards S , Aziz N , Bale S , et al. Standards and guidelines for the interpretation of sequence variants: a joint consensus recommendation of the American College of Medical Genetics and Genomics and the Association for Molecular Pathology. Genet Med 2015;17:405–423. 25741868 10.1038/gim.2015.30 PMC4544753

[13] Xu NJ , Henkemeyer M . Ephrin‐B3 reverse signaling through Grb4 and cytoskeletal regulators mediates axon pruning. Nat Neurosci 2009;12:268–276. 19182796 10.1038/nn.2254 PMC2661084

[14] Perry TL , Bratty PJ , Hansen S , et al. Hereditary mental depression and Parkinsonism with taurine deficiency. Arch Neurol 1975;32:108–113. 1122173 10.1001/archneur.1975.00490440058009

[15] Ohshima S , Tsuboi Y , Yamamoto A , et al. Autonomic failures in Perry syndrome with DCTN1 mutation. Parkinsonism Relat Disord 2010;16:612–614. 20702129 10.1016/j.parkreldis.2010.07.001

[16] Caroppo P , Le Ber I , Clot F , et al. DCTN1 mutation analysis in families with progressive supranuclear palsy‐like phenotypes. JAMA Neurol 2014;71:208–215. 24343258 10.1001/jamaneurol.2013.5100 PMC4169198

[17] Araki E , Tsuboi Y , Daechsel J , et al. A novel DCTN1 mutation with late‐onset parkinsonism and frontotemporal atrophy. Mov Disord 2014;29:1201–1204. 24676999 10.1002/mds.25833

[18] Mishima T , Ishikawa T , Imamura K , et al. Cytoplasmic aggregates of dynactin in iPSC‐derived tyrosine hydroxylase‐positive neurons from a patient with Perry syndrome. Parkinsonism Relat Disord 2016;30:67–72. 27346608 10.1016/j.parkreldis.2016.06.007

[19] Li S , Finley J , Liu ZJ , et al. Crystal structure of the cytoskeleton‐associated protein glycine‐rich (CAP‐Gly) domain. J Biol Chem 2002;277:48596–48601. 12221106 10.1074/jbc.M208512200

[20] Waterman‐Storer CM , Karki S , Holzbaur EL . The p150Glued component of the dynactin complex binds to both microtubules and the actin‐related protein centractin (Arp‐1). Proc Natl Acad Sci USA 1995;92:1634–1638. 7878030 10.1073/pnas.92.5.1634 PMC42574

[21] Ligon LA , Shelly SS , Tokito M , Holzbaur EL . The microtubule plus‐end proteins EB1 and dynactin have differential effects on microtubule polymerization. Mol Biol Cell 2003;14:1405–1417. 12686597 10.1091/mbc.E02-03-0155 PMC153110

[22] Lloyd TE , Machamer J , O'Hara K , et al. The p150(Glued) CAP‐Gly domain regulates initiation of retrograde transport at synaptic termini. Neuron 2012;74:344–360. 22542187 10.1016/j.neuron.2012.02.026 PMC3353876

[23] Moughamian AJ , Holzbaur EL . Dynactin is required for transport initiation from the distal axon. Neuron 2012;74:331–343. 22542186 10.1016/j.neuron.2012.02.025 PMC3347924

[24] McKenney RJ , Huynh W , Vale RD , Sirajuddin M . Tyrosination of alpha‐tubulin controls the initiation of processive dynein‐dynactin motility. EMBO J 2016;35:1175–1185. 26968983 10.15252/embj.201593071 PMC4888239

[25] Nirschl JJ , Magiera MM , Lazarus JE , et al. alpha‐Tubulin tyrosination and CLIP‐170 phosphorylation regulate the initiation of dynein‐driven transport in neurons. Cell Rep 2016;14:2637–2652. 26972003 10.1016/j.celrep.2016.02.046 PMC4819336

[26] Ahmed S , Sun S , Siglin AE , et al. Disease‐associated mutations in the p150(Glued) subunit destabilize the CAP‐gly domain. Biochemistry 2010;49:5083–5085. 20518521 10.1021/bi100235z PMC2938955

[27] Kuźma‐Kozakiewicz M , Chudy A , Kaźmierczak B , et al. Dynactin deficiency in the CNS of humans with sporadic ALS and mice with genetically determined motor neuron degeneration. Neurochem Res 2013;38:2463. 10.1007/s11064-013-1160-7 PMC3898179 24078265

[28] Tanaka F , Ikenaka K , Yamamoto M , Sobue G . Neuropathology and omics in motor neuron diseases. Neuropathology 2012;32:458–462. 22187969 10.1111/j.1440-1789.2011.01281.x

[29] Li K , Yang L , Zhang C , et al. HPS6 interacts with dynactin p150Glued to mediate retrograde trafficking and maturation of lysosomes. J Cell Sci 2014;127:4574–4588. 25189619 10.1242/jcs.141978

[30] Karki S , Holzbaur EL . Affinity chromatography demonstrates a direct binding between cytoplasmic dynein and the dynactin complex. J Biol Chem 1995;270:28806–28811. 7499404 10.1074/jbc.270.48.28806

[31] King SJ , Brown CL , Maier KC , et al. Analysis of the dynein‐dynactin interaction in vitro and in vivo. Mol Biol Cell 2003;14:5089–5097. 14565986 10.1091/mbc.E03-01-0025 PMC284810

[32] Huang N , Lee I , Marcotte EM , Hurles ME . Characterising and predicting haploinsufficiency in the human genome. PLoS Genet 2010;6:e1001154. 20976243 10.1371/journal.pgen.1001154 PMC2954820

[33] Beaudouin J , Gerlich D , Daigle N , et al. Nuclear envelope breakdown proceeds by microtubule‐induced tearing of the lamina. Cell 2002;108:83–96. 11792323 10.1016/s0092-8674(01)00627-4

[34] Chaudhary N , Courvalin JC . Stepwise reassembly of the nuclear envelope at the end of mitosis. J Cell Biol 1993;122:295–306. 8391536 10.1083/jcb.122.2.295 PMC2119651

[35] Yang L , Guan T , Gerace L . Integral membrane proteins of the nuclear envelope are dispersed throughout the endoplasmic reticulum during mitosis. J Cell Biol 1997;137:1199–1210. 9182656 10.1083/jcb.137.6.1199 PMC2132536

[36] Li H , Liu XS , Yang X , et al. Polo‐like kinase 1 phosphorylation of p150Glued facilitates nuclear envelope breakdown during prophase. Proc Natl Acad Sci USA 2010;107:14633–14638. 20679239 10.1073/pnas.1006615107 PMC2930408

[37] Lai C , Lin X , Chandran J , et al. The G59S mutation in p150(glued) causes dysfunction of dynactin in mice. J Neurosci 2007;27:13982–13990. 18094236 10.1523/JNEUROSCI.4226-07.2007 PMC2367233

[38] Feng SY , Li LY , Feng SM , Zou ZY . A novel VRK1 mutation associated with recessive distal hereditary motor neuropathy. Ann Clin Transl Neurol 2019;6:401–405. 30847374 10.1002/acn3.701 PMC6389749

[39] Sedghi M , Moslemi AR , Olive M , et al. Motor neuron diseases caused by a novel VRK1 variant ‐ a genotype/phenotype study. Ann Clin Transl Neurol 2019;6:2197–2104. 31560180 10.1002/acn3.50912 PMC6856620

[40] Lazarus JE , Moughamian AJ , Tokito MK , Holzbaur EL . Dynactin subunit p150(Glued) is a neuron‐specific anti‐catastrophe factor. PLoS Biol 2013;11:e1001611. 23874158 10.1371/journal.pbio.1001611 PMC3712912

[41] Yu J , Lai C , Shim H , et al. Genetic ablation of dynactin p150(Glued) in postnatal neurons causes preferential degeneration of spinal motor neurons in aged mice. Mol Neurodegener 2018;13:10. 29490687 10.1186/s13024-018-0242-z PMC5831668

[42] Ephraim PL , Dillingham TR , Sector M , et al. Epidemiology of limb loss and congenital limb deficiency: a review of the literature. Arch Phys Med Rehabil 2003;84:747–761. 12736892 10.1016/s0003-9993(02)04932-8

[43] Alexander PG , Clark KL , Tuan RS . Prenatal exposure to environmental factors and congenital limb defects. Birth Defects Res C Embryo Today 2016;108:243–273. 27768243 10.1002/bdrc.21140

[44] Basit S , Khoshhal KI . Genetics of clubfoot; recent progress and future perspectives. Eur J Med Genet 2018;61:107–113. 28919208 10.1016/j.ejmg.2017.09.006

[45] Casasnovas C , Cano LM , Albertí A , et al. Charcot‐Marie‐Tooth disease. Foot & Ankle Spec 2008;1:350–354. 10.1177/1938640008326247 19825739

[46] Liao CC , Qi HX , Reed JL , et al. Congenital foot deformation alters the topographic organization in the primate somatosensory system. Brain Struct Funct 2016;221:383–406. 25326245 10.1007/s00429-014-0913-7 PMC4446245

[47] Baets J , Deconinck T , De Vriendt E , et al. Genetic spectrum of hereditary neuropathies with onset in the first year of life. Brain 2011;134(Pt 9):2664–2676. 21840889 10.1093/brain/awr184 PMC3170533

